# Sequential correction using satellite rod for the treatment of severe rigid spinal deformity: a retrospective study of 19 cases

**DOI:** 10.1186/s40001-022-00941-3

**Published:** 2022-12-29

**Authors:** Huipeng Yin, Kun Wang, Shuai Li, Yu Song, Xiaobo Feng, Wenbin Hua, Xinghuo Wu, Yukun Zhang, Cao Yang

**Affiliations:** Department of Orthopedics, Union Hospital, Tongji Medical College, Huazhong University of Science and Technology, Wuhan 430022, China

**Keywords:** Severe rigid spinal deformity, Sequential correction, Satellite rod

## Abstract

**Objectives:**

The purpose of this study was to evaluate the effectiveness of sequential correction using satellite rod in patients with severe rigid spinal deformity undergoing posterior-only PVCR.

**Methods:**

19 patients with severe rigid spinal deformity who underwent PVCR at our center from January 2014 to December 2019 were reviewed. Radiographic measurements, including major coronal Cobb angle, kyphotic curve angle, coronal and sagittal balance were measured. Clinical results were noted, including the SRS-22 questionnaire, the Oswestry Disability Index score, and complications.

**Results:**

Total 19 patients were followed at least 2 years. The mean coronal Cobb angle decreased from 122.7° ± 13.17° to 57.89° ± 8.65° postoperatively, and to 58.42° ± 8.98° at final follow-up. Correction rate is 52.8%. The kyphotic curve angle improved from 102.2° ± 17.05° preoperatively to 39.68° ± 13.67° postoperatively, and to 37.74° ± 12.14° at final follow-up. Correction rate is 61.2%. Compared to preoperative results, apex vertebral translation, ODI and SRS-22 were significantly improved at the final follow-up.

**Conclusions:**

For patients with severe rigid spinal deformities, sequential correction with an auxiliary satellite rod can effectively reduce surgical difficulty and improve correction rate.

## Introduction

Scoliosis naturally progresses to severe rigid spinal deformity because of economic factors, delayed diagnosis, and aggressive patterns, especially in developing countries. Differential diagnosis such as red flags in early stage has been demonstrated to improve prognosis [1, 2]. In general, severe rigid spinal deformity is defined by a major curve magnitude greater than 90 and a flexibility less than 20% [3]. Leaving severe and rigid scoliotic curves untreated may lead to cosmetic concerns, symptoms of pain, neurological deficits and cardiopulmonary impairment, as well as significant morbidity and mortality [4]. The benefits of surgical interventions for improving quality of life and lengthening lifespan are becoming increasingly apparent [5]. However, high-risk patients usually have poor underlying conditions, complications are more likely to occur. The stiff spine places great demands on the surgeon's surgical skills.

It is essential to develop detailed surgical plans based on the curve's location, magnitude, and stiffness prior to surgery. More and more evidences have shown that posterior-only osteotomy and instrumented fusion have excellent surgical outcomes in recent years [6–10]. The posterior vertebral column resection (PVR) involves removing one or more vertebrae to improve trunk balance and curvature correction. PVCR achieves a correction rate between 51% and 61%, according to the literature [11–14]. However, the PVCR's high prevalence of problems, particularly neurological issues, severely restricts its development and application. Initially, Suk et al*.* reported significant complications (34.3%) in PVCR for severe rigid spinal deformity, including two with complete cord injuries [11]. The overall complication rate of 147 patients undergoing VCR was 58.5% according to Lenke et al*.*, including 68 intraoperative complications and 43 postoperative complications [13].

Osteotomy correction is the most common cause of complications. For better surgical outcomes, a safer and more effective method is needed during osteotomy correction. The purpose of this study is to report our experiences with severe rigid spinal deformity patients who underwent sequential correction during PVCR.

## Materials and methods

We reviewed a total of 19 patients with severe rigid spinal deformity who underwent PVCR at our center from January 2014 to December 2019, all of whom were operated on by the senior author. At least 2 years of clinical and radiological follow-up were conducted on these patients. A prior approval was obtained from our Institutional Ethics Committee, as well as informed consent from each patient.

### Preoperative planning

Several radiographs were obtained before surgery, including posterior–anterior and lateral views of full-length spinal radiographs, and bilateral side-bending views. Full-length spinal CT and MRI were obtained to reconstruct 3D spinal deformity and assess the condition of spinal cord and vertebral pedicles. Preoperatively, a detailed surgical strategy was developed based on the patient's symptoms and examination results, including fixation and osteotomy segments.

### Operative technique

**Fig. 1:**
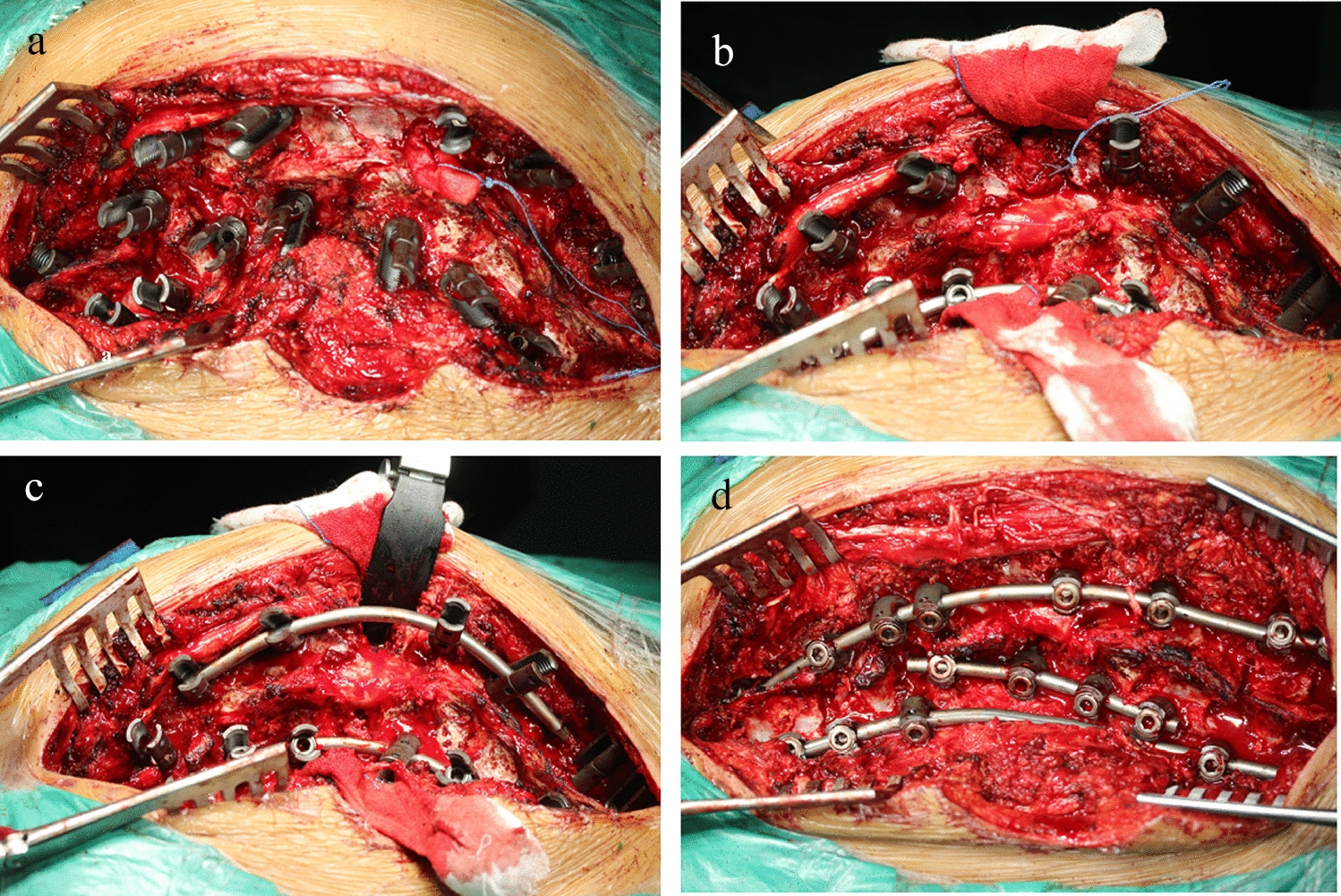
Surgical process diagram. **a** Pedicle screws were implanted; **b** PVCR in the apical region and short rod on the concave side; **c** two short precontoured satellite rod was installed and partial correction; **d** completed correction procedures, and the short rod on the concave side was retained

Under general anesthesia, all surgical corrections were performed via posterior only approach with the patient in a prone position. A posterior midline incision was made and posterior elements were exposed over the entire instrumented area. The costovertebral joints were then dissected in the apical region for PVCR. Pedicle screws were implanted at the levels as planned using free hand technique. Intraoperative radiography of the C-arm was used to determine the optimal position. Using an ultrasonic bone scalpel, PVCR was performed in the apical region, two short precontoured satellite rod was installed on the both side of the apical region and the main curve was gradually reduced by a combined derotation and distraction maneuver. The short rod on the concave side was then retained and locked, and the short rod on the convex side was replaced by a long rod, which is fully engaged in the screw by applying cantilever forces. Another long rod is mounted on the concave side, passing through the top area held by the short rod (Fig. 1). Somatosensory-evoked potentials (SEPs) and motor evoked potentials (MEPs) were monitored throughout the procedure. Drainage tubes were placed in the surgical area, and the wound is closed in layers. Drainage tubes were usually maintained for 48–72 h.

### Data collection

Extensive review of patients’ medical records was performed to determine clinical and radiographic measurements. Demographic and surgical information such as fusion level, pedicle screw placement, curve correction rate, correction loss, estimated blood loss, operative time, and complications were comprehensively recorded. The major coronal Cobb angle, kyphotic curve angle, apex vertebral translation (AVT), coronal and sagittal balance were measured and recorded. Kyphotic curve angle is the maximal amount of kyphosis between the most tilted vertebrae. AVT was measured as the distance from the perpendicular line drawn from the center sacral vertical line to the midpoint of the apical vertebral body. Coronal balance was measured as horizontal distance between the C7 vertebrae and central sacral vertical line. Sagittal balance was measured as the horizontal distance between the C7 vertebrae and the posterior–superior corner of S1. The Oswestry Disability Index (ODI) and SRS-22 questionnaire were assessed before surgery and at the final follow-up.

### Statistical analysis

All data were presented as a mean ± standard deviation. The SPSS (version 18.0, SPSS Inc., Chicago, IL) was used to perform analyses. Paired Student *t* test was used to compare pre-, post-operative and follow-up data. Statistical significance was set at p < 0.05.

## Result

### Patient characteristics and surgery-related data

We retrospectively analyzed 19 patients who underwent PVCR for severe rigid spinal deformity, 8 men and 11 women with an average age of 27.74 ± 13.16 (range 12–56). The demographic characteristics of the study population are shown in Table 1. The mean main coronal Cobb angle was 122.7° ± 13.17° (range 100°–146°), and the mean curve flexibility was 8.37° ± 5.54° (range 0°–19°). The mean operative time was 501 ± 109.3 min (range 380–900 min), with a mean volume of blood loss of 1647 ± 236.6 mL (range 1100–2000). The mean instrumented fusion segment was 13.53 ± 0.96 segments (range 12–15 segments).

**Table 1:** Demographic characteristics of the all study population

Item	Value
Patient number	19 (8 men, 11 women)
Age (years)	27.74 ± 13.16
BMI (kg/m2)	20.64 ± 2.82
mean coronal Cobb angle (°)	122.7 ± 13.17
Mean curve flexibility (°)	8.37 ± 5.54
Mean operative time (minutes)	501 ± 109.3
Mean volume of blood loss (ml)	1647 ± 236.6
Mean fusion segements	13.53 ± 0.96

### Radiographic and clinical results

**Fig. 2:**
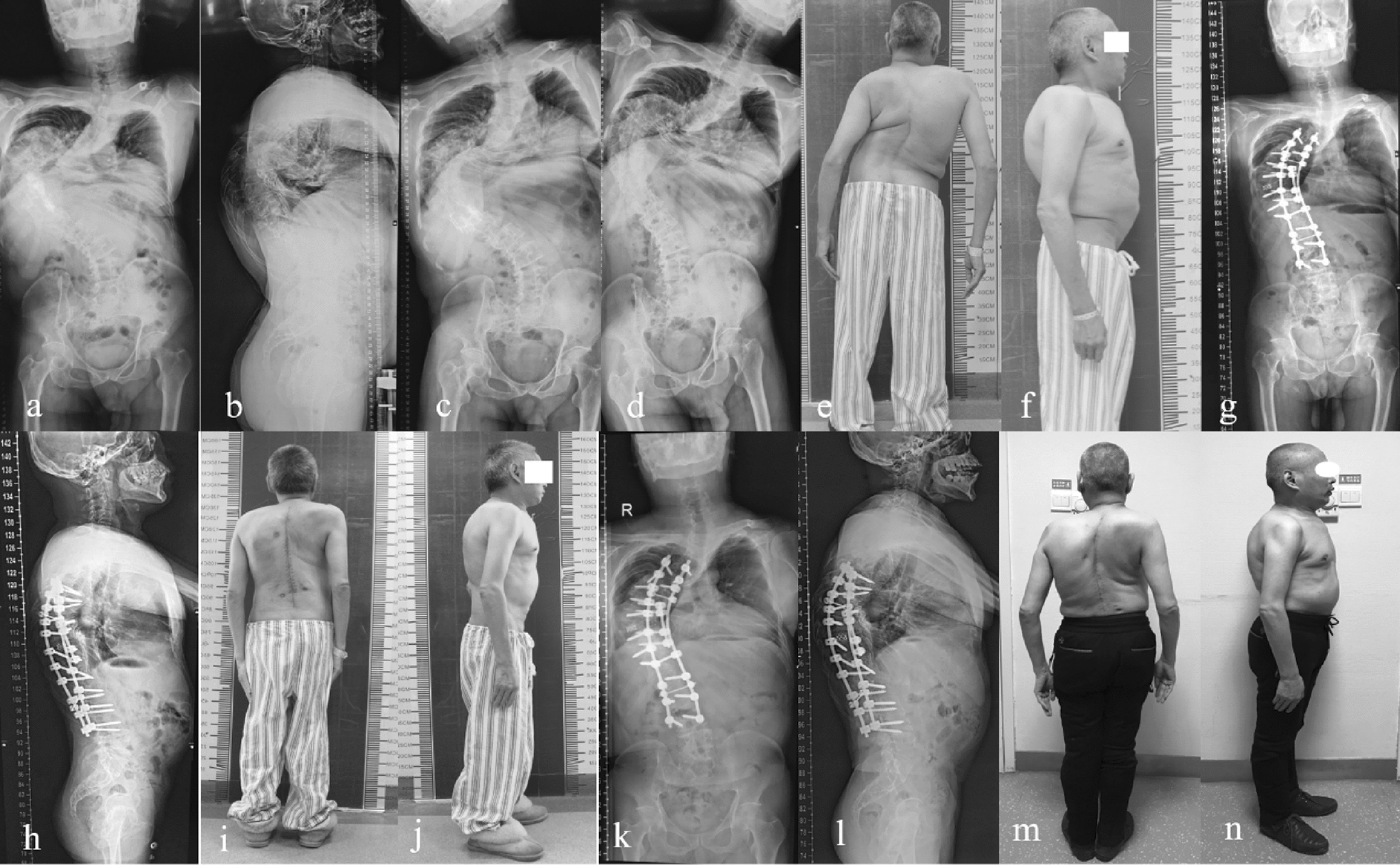
Standing radiographs and images of a 57-year-old female with severe rigid spinal deformity. **a**–**d** Preoperative radiographs; **e**–**f** preoperative appearance in posterior and lateral view; **g**–**h** postoperative anterior–posterior and lateral radiographs; **i**–**j** postoperative appearance in posterior and lateral view; **k**–**l** anterior–posterior and lateral radiographs at the final follow-up; **m**–**n** posterior and lateral appearance at the final follow-up

**Fig. 3:**
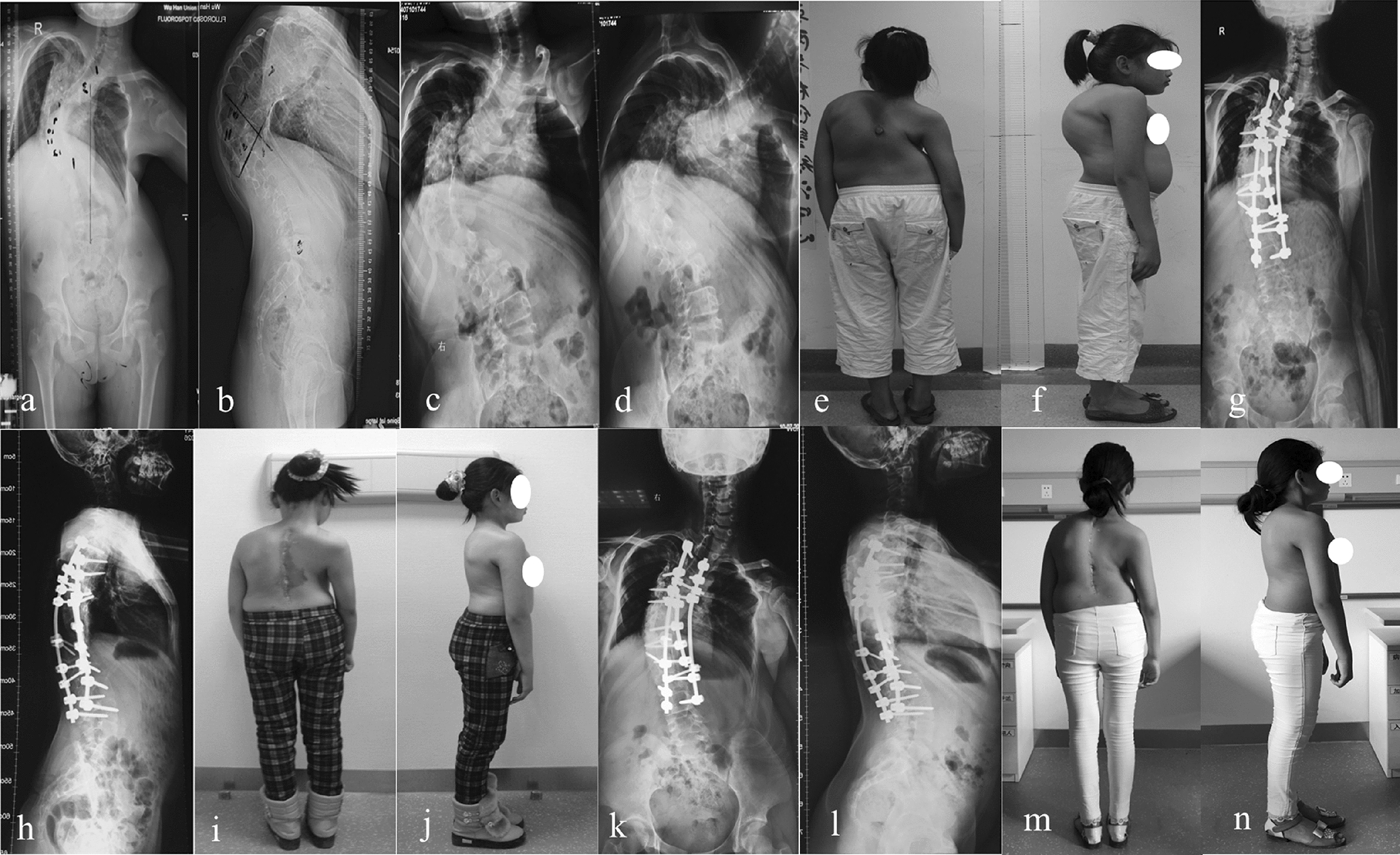
Standing radiographs and images of a 13-year-old female with severe rigid spinal deformity. **a**–**d** Preoperative radiographs; **e**–**f** preoperative appearance in posterior and lateral view; **g**–**h** postoperative anterior–posterior and lateral radiographs; **i**–**j** postoperative appearance in posterior and lateral view; **k**–**l** anterior–posterior and lateral radiographs at the final follow-up; **m**–**n** posterior and lateral appearance at the final follow-up

The mean coronal Cobb angle was decreased to 57.89° ± 8.65° postoperatively, and to 58.42° ± 8.98° at final follow-up. A correction rate of 52.8% were achieved after operation, which only 0.9% loss of correction at final follow-up. The kyphotic curve angle improved from 102.2° ± 17.05° preoperatively to 39.68° ± 13.67° postoperatively, and to 41.26° ± 11.91° at final follow-up. A correction rate of 61.2% were achieved after operation, which only 4.0% loss of correction at final follow-up. The mean preoperative coronal and sagittal imbalances of 32.00 ± 20.68 and 45.99 ± 56.25 mm were changed to 34.37 ± 19.87 and 30.53 ± 25.01 mm at postoperative measurements, respectively. There was no significant difference at final follow-up. The AVT was decreased from 96.16 ± 19.96 mm preoperatively to 56.74 ± 21.91 mm postoperatively, and to 57.00 ± 21.08 mm at final follow-up. The mean ODI score improved from 36.68 ± 11.13 preoperatively to 19.37 ± 6.93 at the final follow-up postoperatively, For the SRS-22 questionnaire, the score was improved significantly at the final follow-up, compared with preoperative scores. (Figs. 2 and 3, Table 2).

**Table 2:** Radiographic and clinical parameters

	Pre-op	Post-op	*p*	Follow-up	*p*
Major coronal Cobb angle (°)	122.7 ± 13.17	57.89 ± 8.65	0.000	58.42 ± 8.98	0.000
Coronal curve correction rate (%)		52.8		52.3	
Kyphotic curve angle (°)	102.2 ± 17.05	39.68 ± 13.67	0.000	37.74 ± 12.14	0.000
Kyphotic curve correction rate(%)		61.2		63.1	
Apex vertebral translation (mm)	96.16 ± 19.96	56.74 ± 21.91	0.000	57.00 ± 21.08	0.000
Coronal balance (mm)	32.00 ± 20.68	34.37 ± 19.87	0.721	34.95 ± 19.51	0.654
Sagittal balance (mm)	45.99 ± 56.25	30.53 ± 25.01	0.280	30.32 ± 23.89	0.271
ODI	36.68 ± 11.13			19.37 ± 6.93	0.001
SRS-22	2.47 ± 0.55			3.37 ± 0.64	0.001

### Complications

Intraoperative SEP–MEP monitoring was abnormal in 8 patients, and neurological complications occurred in 3 of them. These 3 patients had tuberculous kyphotic deformity with severe neurological complications preoperatively. 6 patients developed pneumothorax and were treated with closed thoracic drainage. Cerebrospinal fluid leakage was observed in 4 patients. Wound infection, delayed healing, or nonhealing were not appeared. There was no incidence of superior mesenteric artery syndrome, deep venous thrombosis of the lower extremity, or pulmonary embolism. We did not observe the fixation failure up to the final follow-up.

## Discussion

No specific manifestation of scoliosis in early stage occurs until appearance deformity. Back pain is sometimes a permeance of scoliosis, which leads to the neglect of the diagnosis of scoliosis. Early differential diagnosis to exclude other severe problems is particularly important [15–17]. Poor prognosis is more often appeared in untreated patients with severe rigid spinal deformity, with approximately 5% of them developing chronic respiratory failure as adults [18]. Most notably, not only curvature increase but also rigid changes resulted in the decrease of spinal flexibility. Extremely severe rigid spinal deformity could lead to long-term sequelae of the respiratory system, reduced lung endurance and increased mortality [19]. Therefore, such patients require surgical treatment. For severe rigid spinal deformity, spinal osteotomies with pedicle screws construct are the most option of choice to correct such complicated deformity. However, spinal cord injury, respiratory dysfunction aggravation, even death, plague spine surgeons. In addition to prevent progression of deformity and correct spinal deformity for restoration and reconstruction of trunk balance, prevent nerve injury and pulmonary complications and improve patient’s quality of life are also the important purposes.

To date, there have been many surgical approaches to treat severe spinal deformities, including preoperative traction followed by posterior instrumentation, combined anterior and posterior procedure, PSO or VCR by posterior procedure [7, 10, 20]. PVCR has been widely reported to treat severe rigid spinal deformity. One or more vertebral segments are resected to provide multiplanar deformity correction and maximize correction results of severe spinal deformities. Suk et al*.* first applied PVCR to treat severe scoliosis patients whose major curve was 111° ± 25° with flexibility of 18.2% ± 6.6% and the results showed an immediate main curve correction of 56.4% [11]. Lenke et al*.* reported mean improvements of 61° (51%) in main curve and 56° (55%) in FK for severe pediatric spinal deformities [21]. Furthermore according to a retrospective study of 390 spinal deformity patients, the correction rate of PVCR achieves 63.1% and 61.2% in kyphosis and in scoliosis, respectively [14]. In our study, the correction rate of spinal deformity was 52.8%, which behaved similar to the results reported in the literature.

PVCR has been proved to be the most powerful osteotomy technique to achieve maximum correction in both coronal and sagittal planes. Spine is completely separated into two sections because of three-column vertebral osteotomy during PVCR. Due to the complex spinal structure of patients with severe rigid spinal deformity, complexity of PVCR and difficult screw placement remarkably increase the risk of operative time, blood loss, neurological injury, and potential morbidity [22]. According to the literatures, the overall complication rate of PVCR is 69.2% (range from 23.8% to 100%) [23]. The developing neurological complications is the most concerning for surgeon and patients. Significant complications (24 patients, 34.3%) were reported in the first 70 patients who underwent PVCR procedure by Suk et al., including transient neurological injury in 6 (8.6%) patients and complete cord injuries in 2 (2.8%) patients [11]. In 2013, Lenke et al*.* reviewed 147 pediatric patients with 127 PVCRs and 20 circumferential VCRs, the overall complication rate was 58.5%, including 68 intraoperative and 43 postoperative complications. Although no patients experienced permanent neurological deficits, the incidence of intraoperative neurological events was 27%, and 3% patients experienced transient neurological deficits postoperatively [13]. Intraoperative monitoring changes occurred in 10 patients (22%), and one patient progressed to complete spinal cord injury in the study by Papadapoulus et al*.* [24].

Postoperative neurological complications are closely related to preoperative age, etiology, severity of deformity, angulation rate, spinal cord function classification, intraoperative osteotomy site, osteotomy type, and kyphosis correction rate [22]. Pre-existing neurologic dysfunction is believed to be independent risk factors for neurologic deficits during PVCR procedure [25]. Significant pro-operative nerve injury is accompanied by all patients with postoperative neurological complications before operation. Mechanical spinal cord injury and ischemic injury are considered as the main mechanisms underlying neurological complications. In the apical region, severe angular kyphosis greatly increases the tension of the spinal cord, as a result the spinal cord blood flow around this area is impacted. During surgery, multilevel PVCR, spinal cord traction, excessive shortening, ischemic changes, and displacement of the cut end are important causes of intraoperative nerve injury. PVCR has been modified by many authors to reduce the incidence of neurological complications. Posterior element-maintained PVCR was applied to keep instability of the posterior column during osteotomy [26, 27]. It is worth noting that during surgery, deformity correction rate should not be overemphasized, and obtaining satisfactory deformity correction while maintaining neurological integrity is the best option.

In this study, we used permanent satellite rod to perform the strategy of sequential correction. In our experience, short satellite rod was mounted on the concave side of the apex region, and moderate correction was performed to make the severe main curve smaller and reduce the stress of long rod implantation, and the subsequent two long rods were easier to install. In addition, the presence of a satellite rod, which replaces the spinal cord as a suppositional pivot, can effectively control the translation of the resected end from pivot displacement and reduce the excessive traction or shortening of the spinal cord, thereby avoiding the occurrence of neurological complications [3]. Evidences also show that extra satellite rod provides additional support in the apex region. The use of a multiple rods is a safe, simple, and effective method to provide increased stability across three-column osteotomy sites to significantly prevent implant failure and symptomatic pseudarthrosis [28]. A finite-element study found that the internal fixation structure with satellite rod can improve the overall stability [29]. Furthermore, two rods at the concave site provide twice distraction procedures as a result as greater correction rate [30]. Satellite rods were retained considering the following: (1) pre-bent short rod can play a hinge role in correction, avoid displacement of the cut end, and avoid neurological symptoms; (2) satellite rod facilitate the installation of two long rods and effectively shorten the operation time; (3) preserved satellite rod can avoid pedicle screw loosening caused by repeated fixation and provide additional strong support for the apex region.

We do recognize the drawbacks of our study. A limited number of single center patients were included into this study, statistical analysis might be affected by selection bias. As a result of this retrospective study, surgical skills have advanced over several years and may introduce potential confounding factors and bias. Postoperative CT was not routinely performed, so we could not obtain a more detailed assessment of apical rotation. We will further expand the sample size and extend the follow-up time with a view to obtaining more accurate outcome.

## Conclusions

In this study, we described a novel sequential correction technique for the surgical treatment of severe rigid spinal deformity. Sequential correction with auxiliary satellite rod can effectively prevent the possibility of nerve injury during surgery, reduce the overall difficulty of surgery, achieve a well deformity correction rate and fewer complications.

## Data Availability

All data are available upon requests through the authors.
